# Characterization of pain-, anxiety-, and cognition-related behaviors in the complete Freund's adjuvant model of chronic inflammatory pain in Wistar–Kyoto rats

**DOI:** 10.3389/fpain.2023.1131069

**Published:** 2023-04-11

**Authors:** Mehnaz I. Ferdousi, Patricia Calcagno, Connie Sanchez, Karen L. Smith, John P. Kelly, Michelle Roche, David P. Finn

**Affiliations:** ^1^ Pharmacology and Therapeutics, School of Medicine, University of Galway, Galway, Ireland; ^2^ Centre for Pain Research, University of Galway, Galway, Ireland; ^3^ Galway Neuroscience Centre, University of Galway, Galway, Ireland; ^4^ Physiology, School of Medicine, University of Galway, Galway, Ireland; ^5^ Alkermes Inc., Waltham, MA, United States

**Keywords:** chronic pain, stress, anxiety, cognition, complete Freund's adjuvant, Wistar–Kyoto rats

## Abstract

**Introduction:**

Chronic pain is often associated with comorbid anxiety and cognitive dysfunction, negatively affecting therapeutic outcomes. The influence of genetic background on such interactions is poorly understood. The stress-hyperresponsive Wistar–Kyoto (WKY) rat strain, which models aspects of anxiety and depression, displays enhanced sensitivity to noxious stimuli and impaired cognitive function, compared with Sprague–Dawley (SD) counterparts. However, pain- and anxiety-related behaviors and cognitive impairment following induction of a persistent inflammatory state have not been investigated simultaneously in the WKY rats. Here we compared the effects of complete Freund's adjuvant (CFA)-induced persistent inflammation on pain-, negative affect- and cognition-related behaviors in WKY vs. SD rats.

**Methods:**

Male WKY and SD rats received intra-plantar injection of CFA or needle insertion (control) and, over the subsequent 4 weeks, underwent behavioral tests to assess mechanical and heat hypersensitivity, the aversive component of pain, and anxiety- and cognition-related behaviors.

**Results:**

The CFA-injected WKY rats exhibited greater mechanical but similar heat hypersensitivity compared to SD counterparts. Neither strain displayed CFA-induced pain avoidance or anxiety-related behavior. No CFA-induced impairment was observed in social interaction or spatial memory in WKY or SD rats in the three-chamber sociability and T-maze tests, respectively, although strain differences were apparent. Reduced novel object exploration time was observed in CFA-injected SD, but not WKY, rats. However, CFA injection did not affect object recognition memory in either strain.

**Conclusions:**

These data indicate exacerbated baseline and CFA-induced mechanical hypersensitivity, and impairments in novel object exploration, and social and spatial memory in WKY vs. SD rats.

## 1. Introduction

Chronic pain (defined as pain that persists or recurs for more than 3 months) is a major unmet clinical problem of current times, causing significant emotional distress and impairing quality of life, work, and daily functioning (1). Being one of the leading causes of disability worldwide, chronic pain has a massive individual, economic, and societal burden (2). Despite the high prevalence of chronic pain, the current treatment strategies are limited, and our understanding of the pathophysiology of chronic pain remains poor (3). Moreover, stress-related affective disorders (anxiety and depression) (4, 5) and cognitive dysfunction (6, 7) are highly comorbid with chronic pain, further contributing to the debilitating nature of the disorder and imposing a huge need for effective treatment.

Chronic pain can arise from persistent inflammation (8). Localized inflammatory responses are commonly modelled preclinically by injecting a noxious chemical such as formalin, carrageenan, or complete Freund's adjuvant (CFA) into the rodent paw (9). Of these, the CFA-induced inflammation lasts for at least 3 weeks, allowing for relatively long-term studies of inflammation-related persistent pain responses (10). Injecting CFA into the rodent paw not only results in mechanical and thermal (heat and cold) hypersensitivity, but also produces place avoidance, mimicking the emotional-affective component of pain (11–13). In addition, anxiety-related behaviors (14, 15) and impairments in multiple cognitive domains (6) have been reported in several rodent models of inflammatory pain, including the CFA model.

The Wistar–Kyoto (WKY) rat is an inbred strain that was originally bred as a normotensive control strain for spontaneously hypertensive rats (16), but later suggested to model several behavioral and neurochemical aspects of anxiety and depression, compared to other rat strains such as Sprague–Dawley (SD) or Wistar (17–20). The WKY rats also exhibit social avoidance, diminished activity in a novel environment, and impaired cognitive function in several behavioral tests (21, 22). Moreover, the WKY rats show heightened stress-induced behavioral and neuroendocrine responses (20, 23). In addition, the WKY rats display exaggerated sensitivity to noxious heat, visceral, and inflammatory (formalin) stimuli, compared to SD rats (17, 24–26). Hence, the WKY rat is of interest for studying the neurobiology underlying pain-cognition/negative affect interactions and the influence of genetic background thereon. Some studies have reported increased mechanical hypersensitivity and depression-like behavior in the WKY rats following peripheral nerve injury (27) and CFA-induced temporomandibular joint inflammation (28), suggesting an influence of genotype on nociceptive and depression-related behaviors in chronic pain. Therefore, we hypothesized that the effects of CFA-induced persistent inflammation on behavioral domains related to nociception, negative affect, and cognition will be exaggerated in the WKY rat strain, compared to SD counterparts. The aims of the present study were to assess sensory and affective components of pain following hind paw CFA injection and examine any associated anxiety-related behaviors and cognitive impairment in WKY vs. SD rats.

## 2. Materials and methods

### 2.1. Animals

Male WKY and SD rats (7–8 weeks old, 180–230 g on arrival; Envigo, UK) were maintained under standard controlled conditions (temperature 21 ± 2°C, humidity 45%–55%, and 12:12 h light/dark cycle with lights on at 07:00 h) throughout the study. Upon arrival, all animals were housed in groups of 3–4 per cage in plastic bottom cages (45 cm × 20 cm × 20 cm) containing 3Rs bedding (>99% recycled paper; Fibrecycle Ltd, UK) and sizzle nest material (LBS Biotechnology, UK). After 5 days of acclimatization, the animals were housed singly in cages for the rest of the study. An additional cohort of male SD and WKY rats (*n* = 16 per strain) were used in this study as conspecifics for the three-chamber sociability test. The conspecific rats were pair-housed by strain for the duration of the study. Food (14% protein rodent diet; Envigo, UK) and water were available *ad libitum*.

All experimental procedures were approved by the Animal Care and Research Ethics Committee, University of Galway. The work was carried out under license (AE19125/P028) from the Health Products Regulatory Authority in the Republic of Ireland and in accordance with EU Directive 2010/63/EU and ARRIVE guidelines.

### 2.2. Complete Freund's adjuvant injection

To induce a chronic inflammatory pain state, immunogenic CFA (catalogue#F5881, Sigma-Aldrich, Ireland) was used (29). CFA consists of heat-killed and dried *Mycobacterium tuberculosis* that is suspended in a mixture of paraffin oil and mannide monooleate, with the 3 constituents contributing to the adjuvant effects. The rats were injected once with 100 µl CFA (1 mg/ml) in the plantar surface of the right hind paw under brief isoflurane anesthesia (2%–3% in 0.8 L/min O_2_). The control animals received an intraplantar needle insertion into the right hind paw under the same conditions. Since we injected the CFA suspension without reconstituting or diluting it in a vehicle, we opted for an intraplantar needle insertion in the sham animals instead of a vehicle injection as the latter will not represent a true control treatment. Intraplantar needle insertion has been used previously as a control for the CFA model (30, 31). After recovery, animals were returned to their home cages.

### 2.3. Study design

**Figure 1:**
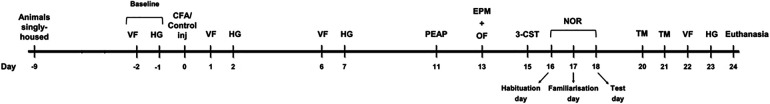
Schematic of the study outlining the timeline of behavioral tests. 3-CST: three-chamber sociability test, CFA, complete Freund's adjuvant; EPM, elevated plus maze; HG, Hargreaves’ test; inj, injection; NOR, novel object recognition test; OF, open field; PEAP, place escape/avoidance paradigm; TM, T-maze test; VF, von Frey test.

The experimental timeline is illustrated in Figure 1. Baseline nociceptive responses to cutaneous mechanical and heat stimuli were assessed in the von Frey and Hargreaves' tests, respectively, for each rat. The rats were then randomly assigned to a treatment condition (control or CFA injection), resulting in 4 experimental groups (*n* = 10 per group): SD-control, SD-CFA, WKY-control, and WKY-CFA. Following CFA/control injections on Day 0, the animals underwent a series of behavioral investigations: (1) mechanical hypersensitivity was assessed using von Frey test on Days 1, 6, and 22; (2) heat hypersensitivity was determined using Hargreaves’ test on Days 2, 7, and 23; (3) place escape/avoidance paradigm evaluated the affective component of pain on Day 11; (4) anxiety-related behavior and general locomotor activity were assessed on Day 13 using elevated plus maze and open field tests; and (5) different cognitive domains were evaluated using the three-chamber sociability test (Day 15), novel object recognition test (Day 18), and T-maze spontaneous alternation task (Days 20–21).

### 2.4. Behavioral testing

For all experimental procedures, the light intensity was measured at the base of the arena. The arenas were cleaned thoroughly between animals with warm soapy water, unless otherwise stated.

#### 2.4.1. von Frey test

The rats were placed in individual Perspex compartments (14 cm × 20 cm × 25 cm per compartment) on a raised wire-mesh floor and allowed to acclimatize for 15 min. The von Frey (VF) test was carried out following the up-and-down method described previously (32, 33) with a series of calibrated nylon filaments (Touch Test Sensory Evaluator #58011, Stoelting, United States) starting with the 2 g filament. Each filament was applied once perpendicular to the plantar surface of the hind paw targeting the area at the base of the third and fourth digits (from medial to lateral). A sufficient uniform force was applied to cause slight buckling of the filament for approximately 5 s. In case of a positive response (flinching, licking, or a brisk withdrawal of the paw), a lower weight filament was tested. In case of a negative response, a higher weight filament was tested. After the first change in response pattern in either case (i.e., from no response to response or vice-versa), four additional stimulations were performed. In all cases, a maximum of 9 stimulations was applied to each paw. The response pattern is associated to a constant, *k*, in the table by Dixon (32). The withdrawal threshold (g) for each paw was then calculated using the following formula:

$$\mathrm{Paw}\;\mathrm{Withdrawal}\;\mathrm{Threshold}\;\;(\mathrm{PWT})\;=\;10^{\left[\log\;(\mathrm{last}\;\mathrm{filament})\;+\;0.3k\right]}$$

#### 2.4.2. Hargreaves' test

The Hargreaves' (HG) test apparatus (IITC Life Sci Inc, United States) consisted of six clear Perspex compartments (11 cm × 20 cm × 15 cm per compartment) placed on top of an elevated glass panel maintained at 30 ± 1°C. After acclimatizing the rats to the arena for 15 min, a focused beam of radiant light (active intensity of 30% corresponding to 53°C) was applied from below to the plantar surface of the hind paw (same location as for the VF filaments) for up to 20 s. The latency to flinch, lick or briskly withdraw the hind paw was recorded. The stimulus was repeated four times on each hind paw with an interval of at least 5 min, and the average withdrawal latency (s) for each paw was then calculated.

#### 2.4.3. Place escape/avoidance paradigm

The place escape/avoidance paradigm (PEAP) measures and dissociates the affective/motivational and sensory components of pain processing (34). The test was conducted on Day 11 post-injection as described previously (12). Briefly, a clear Perspex arena (30 cm × 30 cm × 30 cm) was placed on top of a raised wire mesh and divided into two compartments by a central partition with a small opening. One compartment (light side, 20–22 lux) was transparent and the other compartment (dark side, 0–1 lux) was covered with black paper and had a wooden black lid on top. The animals were always placed into the light side facing away from the central opening/dark side. The rats were acclimatized to the arena for 30 min one day prior to testing, and on the test day for an additional 10 min. Testing began immediately thereafter with the animal receiving a noxious mechanical stimulation using a suprathreshold VF filament (60 g) to the plantar surface of the hind paw (same location as for the VF test) at an interval of 15 s for 30 min. During this 30-min trial, the rat was allowed unrestricted movement throughout the arena. The ipsilateral (i.e., CFA-injected) paw was stimulated when the animal was in the dark side and the contralateral paw was stimulated when it was in the light side. This sets up a situation in which the animal has a choice to move to the aversive light (non-preferred) side to escape/avoid noxious mechanical stimulation to the injured area in the dark (normally preferred) side. The animal was considered to be in a given side of the arena where both its hind paws were located. The behavioral responses to noxious stimuli (criteria for a positive response was like those used for VF test) were recorded manually during the trial. Activity of the animals in the light side was used as an index of avoidance behavior. The behaviors (time spent and number of entries into the light side) of each animal were recorded and later scored using the Ethovision XT 11.5 software (Noldus, Netherlands).

#### 2.4.4. Elevated plus maze test

On Day 13 post-injection, the animals were assessed for anxiety-related behavior in the elevated plus maze (EPM) test. The wooden arena, which was elevated 50 cm above the floor, consisted of a central platform (10 cm × 10 cm) connecting four arms (each arm: 50 cm × 10 cm), of which two arms were enclosed by walls (30 cm high, 25 lux) and two arms with no enclosure (60 lux). On the test day, the animals were placed in the center zone of the maze with their heads facing an open arm, and the behaviors were recorded for 5 min with a video camera positioned on top of the arena. The Ethovision software was used later to score the time spent in each zone.

#### 2.4.5. Open field test

Following the EPM test, rats were immediately exposed to the open field (OF) test. The circular arena consisted of a floor (75 cm diameter) and aluminum walls (40 cm high). The animals were placed in the center of the novel open environment that was brightly lit (280–300 lux). A video camera above the arena recorded the behaviors for 5 min. Using Ethovision system, the total distance moved (cm) in the arena for each rat was later tracked to assess general locomotor activity. The time spent in the center of the arena (40 cm diameter) was also measured as an index of anxiety-related behavior.

#### 2.4.6. Three-chamber sociability test

On Day 15 post-injection, the three-chamber sociability test (3-CST) was conducted to assess social approach behavior and social memory (35). The arena (90.5 cm × 46 cm × 40 cm, 30–40 lux) consisted of three communicating chambers separated by clear Perspex walls with central openings that allowed free access to all chambers. The test consisted of 3 consecutive sessions. Briefly, animals were placed into the center of the arena and allowed to freely explore the empty apparatus for 10 min (habituation phase). The test rat was then allowed to explore the arena with a confined novel conspecific rat (C1) and a novel metal cage placed in each of the outer chambers for another 10 min (sociability phase). The placement of the first conspecific rat into the outer left or right chamber was randomly allocated for each trial to avoid a side preference and counterbalanced within a treatment group. In the third session, the metal cage was replaced with a second confined novel conspecific rat (C2). The test rat was then allowed to freely explore and interact with the first conspecific (now familiar, C1) and the second conspecific (novel, C2) rat for a further 10 min (social novelty preference phase). The conspecifics used for each trial were of the same strain as the test rat. All behaviors were recorded and the Ethovision system was used later to manually score social interactions defined as times spent engaging in investigatory behaviors (sniffing, rearing/climbing against the novel cage, and active interaction with conspecific animals).

#### 2.4.7. Novel object recognition test

The novel object recognition (NOR) test is a measure of recognition memory and primarily relies on the natural tendency of the animal to spend more time exploring new object rather than a previously encountered one (36). The test was performed according to protocols described previously (37, 38). Testing was conducted in the same circular arena used for OF test but with a reduced light intensity (100 ± 10 lux). The objects used were 500 ml transparent plastic Coca-Cola® Zero bottles (filled with water) and an abstract plastic structure of similar dimensions constructed using green, white, and blue toy blocks (Playskool Clipo™ blocks). In all cases, the objects had no apparent natural significance to the rats. The base of the objects was secured to the floor of the arena with adhesive so that the animal could not displace them.

The NOR test consisted of 3 sessions conducted on consecutive days (Days 16–18 post-injection). On the first (habituation) day, animals freely explored the empty arena for 10 min. On the second (familiarization) day, three identical objects (Coca-Cola® Zero bottles) were placed in the arena, approximately 20 cm apart and 15 cm from the arena walls. The rat was placed in the center of the arena facing away from the objects and allowed to freely explore the arena and objects three times for 5 min, with 5 min inter-trial intervals. On the third (test) day, one of the three objects in the arena was replaced with a novel object (toy blocks). The animal was re-exposed to the arena and objects once for 5 min, allowed to explore freely, and then returned to the home cage. The arena and objects were cleaned between each exposure using 70% ethanol to remove any odor and olfactory cue. The position of the novel object was alternated between rats to minimize any orientation bias. All behaviors were recorded and the Ethovision system was used later to manually score the times spent exploring the objects that included sniffing the object, rearing against the object, or having the head directed towards the object within 2 cm of the object. A discrimination index, defined as the time spent exploring the novel object vs. the familiar objects on the test day, was calculated as:

$$\mathrm{Discrimination}\;\mathrm{index}\;=\;\frac{\left(\mathrm{Tim}e_{\mathrm{novel}\;\mathrm{obejct}}\;-\mathrm{Average}\;\;\mathrm{tim}e_{\;\mathrm{familiar}\;\mathrm{objects}}\right)}{\left(\mathrm{Tim}e_{\mathrm{novel}\;\mathrm{object}}+\mathrm{Average}\;\mathrm{tim}e_{\;\mathrm{familiar}\;\mathrm{objects}}\right)}$$

#### 2.4.8. T-maze test

The T-maze spontaneous alternation task was used to assess spatial memory on Days 20 and 21 post-injection. The task is based on the natural tendency of rodents to explore novel environments, that is, to visit a new arm of the maze rather than a familiar arm upon subsequent exposure. The test was performed according to protocols described previously (39). Testing was carried out in a black wooden arena (18–20 lux) in the shape of a “T” that was elevated 40 cm above the floor of the testing room and enclosed by walls of height 30 cm. The maze was set up for testing with the central partition in place and guillotine doors removed from the entrance of the two goal arms. The animal was placed in the start area and after choosing a goal arm (sample phase), the guillotine door was slid down to confine the animal to that arm for 30 s. The central partition was then removed, and the animal was returned to the home cage. After raising both the guillotine doors, the rat was returned to the start area of the maze and again allowed to choose any of the two open goal arms (choice phase). In the choice phase, if the rat chose the alternate arm this was counted as “alternation”, while if it moved to the same arm as sample phase this was considered as an “error in alternation”. The criterion for arm selection in both phases was that the entire animal (whole body plus the tail tip) be on a goal arm. A cut-off of 90 s was set for selecting a goal arm. If the rat failed to select an arm within 90 s in either phase, it was removed from the maze, and this was counted as an “error of omission”. Each rat received 10 trials (5 trials per day). The mean percentages of (1) alternation (2), error in alternation, and (3) omission of trials were then calculated. The maze was cleaned thoroughly between exposures using 70% ethanol to remove any odor and olfactory cue.

### 2.5. Statistical analysis

The data were analyzed using IBM SPSS Statistics 24 (SPSS Inc, United States). The normality and homogeneity of variance were checked using Shapiro–Wilk and Levene's tests, respectively. The time course behavioral data were analyzed with repeated measures analysis of variance (ANOVA) with strain and CFA injection as between-subjects factors and time as within-subjects factor. Sphericity of the datasets for repeated measures ANOVA was checked with Mauchly's Test for Sphericity; if this assumption was violated, a Greenhouse-Geisser correction was used. Other behavioral data were analyzed using two-way ANOVA with strain and CFA injection as factors. *Post hoc* Student–Newman–Keuls (SNK) test was carried out for pairwise comparisons, where appropriate. To assess any effect of differences in locomotor activity between the two strains, two-way analysis of covariance (ANCOVA) was used to analyze behavioral data from 3-CST and NOR tests with distance moved as covariate. In all cases, *p *< 0.05 was considered statistically significant. If the data were not normally distributed and/or the variance was not homogeneous, three transformations were applied in the order: square root, natural logarithm, and ranking of the data values to evaluate if parametric statistics could be used. If any dataset was ordinal (e.g., VF test data) or did not pass assumptions of parametric analysis (even after transformation), non-parametric analysis was performed using Kruskal–Wallis (KW) test followed by *post hoc* Mann–Whitney *U*-test with Bonferroni–Holm correction, where appropriate. Friedman's test followed by Wilcoxon signed-rank test was employed to assess the time course data of VF test.

All graphs were constructed using GraphPad Prism 8.0 (GraphPad Software Inc, United States). Depending on the statistical approach undertaken (parametric or non-parametric, respectively), results were expressed as individual data points with either mean ± standard error of the mean (SEM) or as median with interquartile range (IQR).

## 3. Results

### 3.1. CFA induced greater mechanical hypersensitivity in WKY rats than SD rats

**Figure 2:**
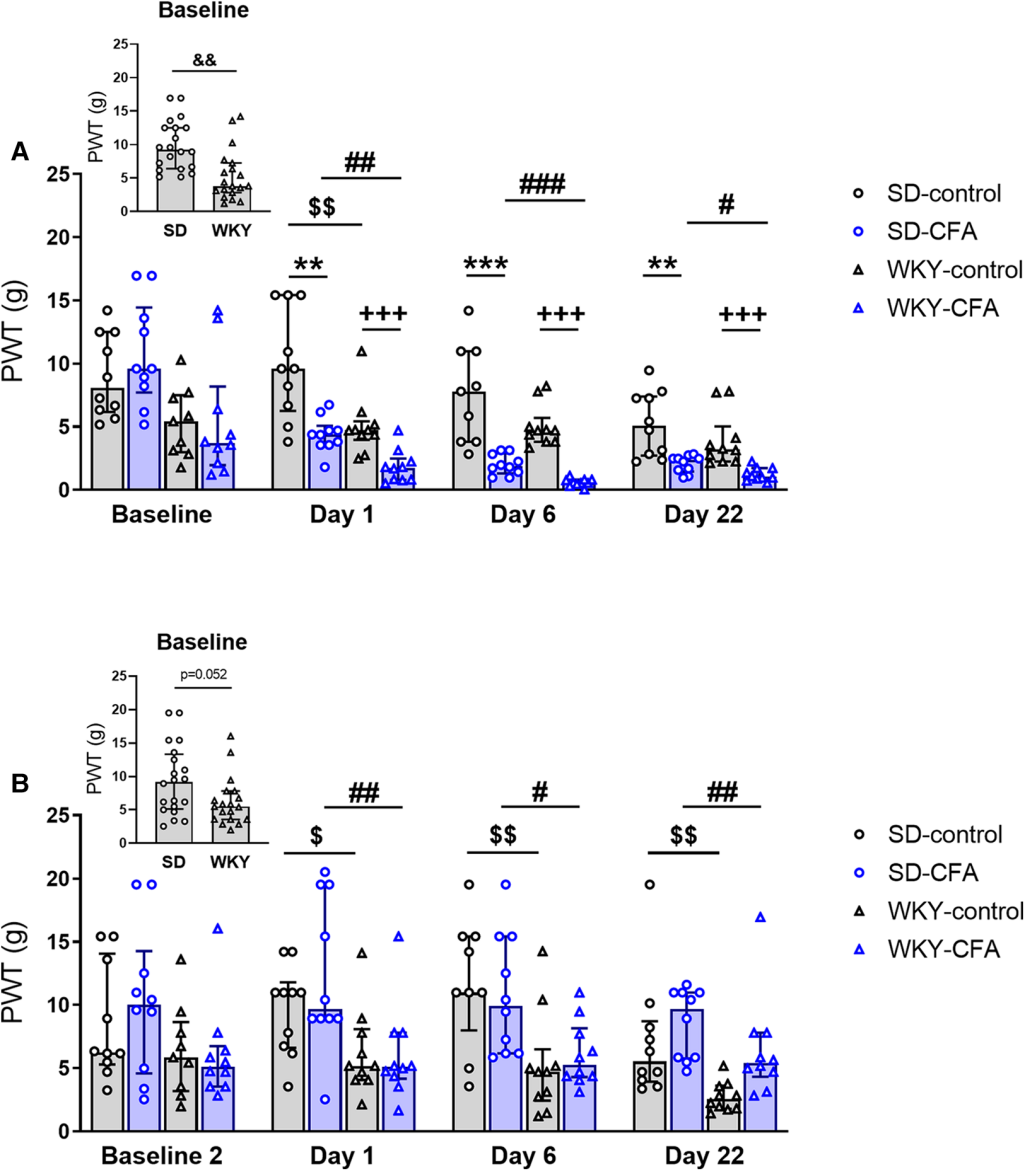
Time course of the effects of CFA injection on nociceptive responding to mechanical stimuli on the (**A**) ipsilateral and (**B**) contralateral hind paws in SD and WKY rats. (**A**) Ipsilateral side: CFA injection produced mechanical hypersensitivity in both rat strains, assessed using VF test [**p < 0.01, ***p < 0.001 (SD-CFA vs. SD-control), ^+++^*p *< 0.001 (WKY-CFA vs. WKY-control)]. CFA-induced mechanical hypersensitivity was greater in WKY rats than SD rats on Days 1, 6, and 22 post-injection [^#^*p *< 0.05, ^##^*p *< 0.01, ^###^*p *< 0.001 (WKY-CFA vs. SD-CFA)]. WKY rats also displayed lower PWT than SD rats at baseline (inset: ^&&^*p *< 0.01, WKY vs. SD, *n* = 20/group) and on Day 1 (^$$^*p *< 0.01, WKY-control vs. SD-control) in the absence of CFA. (**B**) Contralateral side: WKY rats overall showed enhanced mechanical sensitivity compared to SD rats on Days 1, 6, and 22 [^$^*p *< 0.05, ^$$^*p *< 0.01, ^$$^*p *< 0.01 (WKY-control vs. SD-control); ^##^*p *< 0.01, ^#^*p *< 0.05, ^##^*p *< 0.01 (WKY-CFA vs. SD-CFA)]. Data are expressed as median with IQR, *n* = 8–10/group. CFA, complete Freund's adjuvant; PWT, paw withdrawal threshold; SD, Sprague–Dawley; VF, von Frey; WKY, Wistar–Kyoto.

To assess the sensitivity to a mechanical stimulus in SD and WKY rats, paw withdrawal response was measured using the VF test. Friedman's test revealed significant differences in ipsilateral PWT among the experimental groups [*χ*^2^(3) = 37.589, *p *< 0.001]. *Post hoc* Wilcoxon test showed that the withdrawal thresholds on Days 1, 6, and 22 were lower than on baseline in CFA-injected, but not in control, SD and WKY rats (*p *< 0.001), thus indicating mechanical hypersensitivity of the hind paw injected with CFA in both strains (Figure 2A). Moreover, KW analysis on each time point revealed significant main effects on withdrawal thresholds at baseline [*χ*^2^(3) = 9.816, *p *< 0.05], Day 1 [*χ*^2^(3) = 24.447, *p *< 0.001], Day 6 [*χ*^2^(3) = 31.852, *p *< 0.001], and Day 22 [*χ*^2^(3) = 24.453, *p *< 0.001]. *Post hoc* tests showed that CFA-injected SD and WKY rats exhibited lower PWT, compared to respective control counterparts, at all three post-CFA time points (Days 1, 6, and 22: SD-CFA vs. SD-control, *p *< 0.01; WKY-CFA vs. WKY-control, *p *< 0.001), further confirming CFA-induced mechanical hypersensitivity in both strains. In addition, the WKY-CFA rats displayed greater hypersensitivity to evoked mechanical stimulation than SD counterparts at all three post-CFA time points [WKY-CFA vs. SD-CFA: *p *< 0.01 (Day 1), *p *< 0.001 (Day 6), *p *< 0.05 (Day 22); Figure 2A]. Furthermore, WKY rats showed lower PWT than SD rats in the absence of CFA injection at baseline [WKY vs. SD: 4.09 (2.91–7.60) g vs. 9.26 (6.42–12.51) g, *p *< 0.01] and on Day 1 (WKY-control vs. SD-control, *p *< 0.01), but not at later time points.

On the contralateral side (Figure 2B), Friedman's test showed a significant main effect on PWT among experimental groups [*χ*^2^(3) = 10.644, *p *< 0.05] but no differences in PWT between baseline and post-CFA time points were found between relevant groups in the *post hoc* test. In the KW test, significant main effects on contralateral PWT at Day 1 [*χ*^2^(3) = 11.936, *p *< 0.01], Day 6 [*χ*^2^(3) = 13.883, *p *< 0.01], and Day 22 [*χ*^2^(3) = 19.816, *p *< 0.001] were observed. Further *post hoc* analysis indicated that the WKY rats, in comparison to SD rats, displayed lower PWT on the contralateral side (Day 1: WKY-control vs. SD-control, *p *< 0.05; WKY-CFA vs. SD-CFA, *p *< 0.01. Day 6: WKY-control vs. SD-control, *p *< 0.01; WKY-CFA vs. SD-CFA, *p *< 0.05; Day 22: WKY-control vs. SD-control, *p *< 0.01; WKY-CFA vs. SD-CFA, *p *< 0.01). These results indicate an enhanced sensitivity to mechanical stimuli in the WKY rat strain in the absence of any injury.

### 3.2. CFA induced similar heat hypersensitivity in both WKY and SD rats

**Figure 3:**
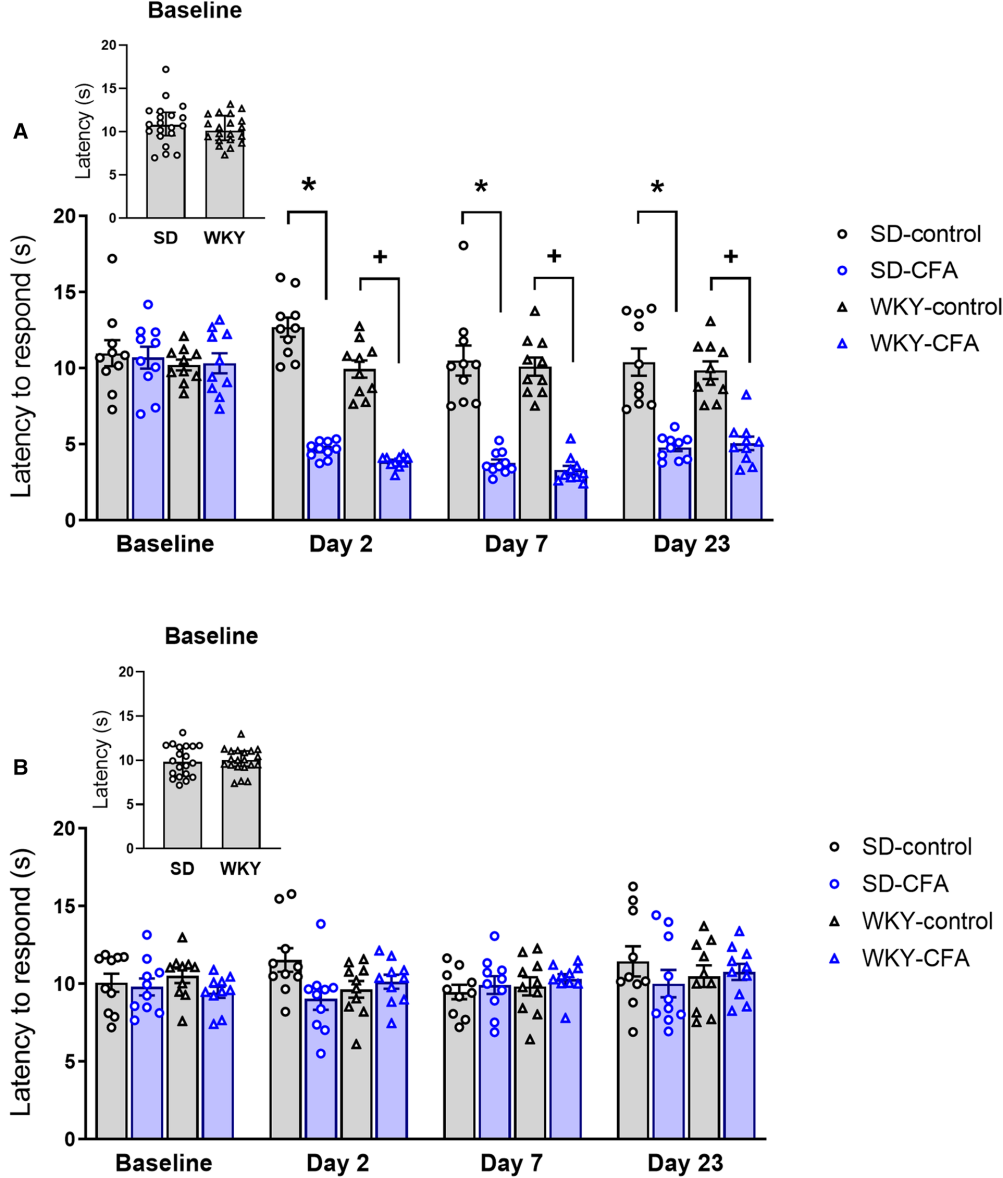
Time course of the effects of CFA injection on nociceptive responding to noxious heat stimuli on the (**A**) ipsilateral and (**B**) contralateral hind paws in SD and WKY rats. (**A**) Ipsilateral side: CFA injection produced similar heat hypersensitivity in SD and WKY rats (assessed in HG test) on Days 2, 7, and 23 post-injection [*p < 0.05 (SD-CFA vs. SD-control), ^+^*p *< 0.05 (WKY-CFA vs. WKY-control)]. (**B**) Contralateral side: Response latency to noxious heat stimulus was similar between WKY and SD rats. Data are expressed as mean ± SEM, *n* = 10/group. CFA, complete Freund's adjuvant; HG, Hargreaves’; PWT, paw withdrawal threshold; SD, Sprague–Dawley; WKY, Wistar–Kyoto.

To assess the sensitivity to a noxious heat stimulus in SD and WKY rats, paw withdrawal latency was measured using the HG test. Repeated measures ANOVA revealed significant effects of time (F_4,144 _= 63.904, *p *< 0.001), time x strain (F_4,144 _= 2.744, *p *< 0.05), and time × CFA (F_4,144 _= 45.736, *p *< 0.001) interactions in tests of within-subjects effects on response latency to noxious heat stimulus on the ipsilateral side. A significant overall effect of CFA (F_1,36 _= 97.396, *p *< 0.001) was also observed in tests of between-subjects effects on response latency. *Post hoc* analysis revealed that the CFA-injected SD and WKY rats exhibited lower response latency, compared to respective control counterparts, at all three post-CFA time points (Days 2, 7, and 23: SD-CFA vs. SD-control, *p *< 0.05; WKY-CFA vs. WKY-control, *p *< 0.05), thus indicating that CFA induced heat hypersensitivity in both strains (Figure 3A). No differences in latency to respond to the evoked heat stimulation were observed between WKY-control and SD-control, or between WKY-CFA and SD-CFA.

On the contralateral side (Figure 3B), repeated measures ANOVA showed significant effects of time (F_3.261,117.392 _= 2.619, *p *< 0.05) and time x strain × CFA interaction (F_3.261,117.392 _= 2.724, *p *< 0.05) in tests of within-subjects effects on response latency. However, *post hoc* analysis showed there was no significant difference between relevant experimental groups.

### 3.3. Neither SD nor WKY rats injected with CFA exhibited avoidance-like behavior in the PEAP

The PEAP was employed to assess the aversive component of pain in SD and WKY rats in the CFA-induced persistent inflammatory pain model. During the test, the CFA-injected (ipsilateral) hind paw was stimulated with a 60 g filament when the animal was in the dark side of the arena. We first analyzed this withdrawal response data. Repeated measures ANOVA revealed a significant main effect of CFA (F_1,35 _= 70.004, *p *< 0.001) on percentage positive paw withdrawal response. Further *post hoc* tests revealed that the CFA-injected SD and WKY rats showed higher percentage positive paw withdrawal response to noxious mechanical stimulation, compared to respective control counterparts, throughout the 30-min testing period [T_0–30_ (in 5 min bins): SD-CFA vs. SD-control, *p *< 0.05; WKY-CFA vs. WKY-control, *p *< 0.05]. This result confirmed that both strains displayed CFA-induced mechanical hypersensitivity during the test.

**Figure 4:**
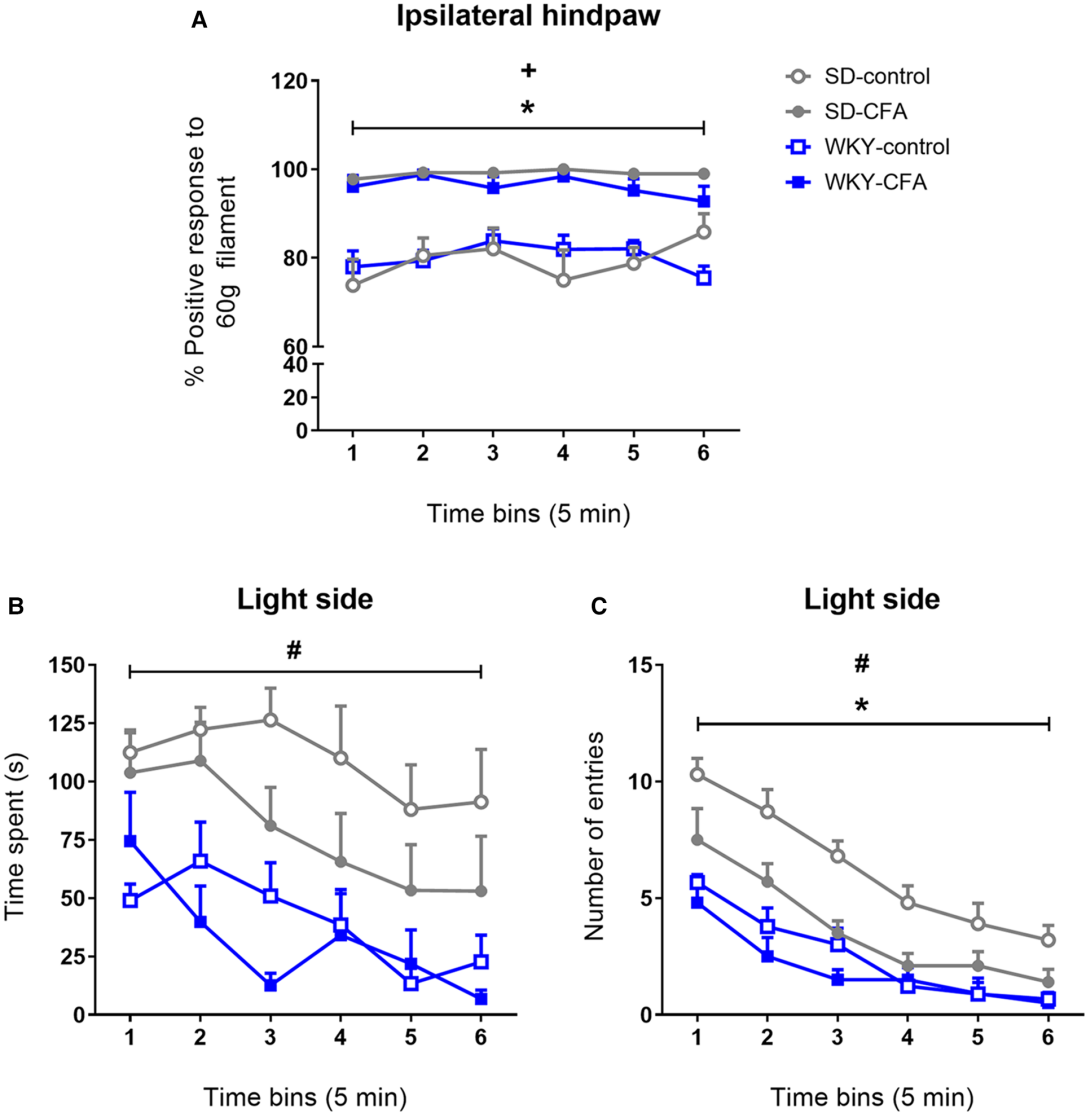
Effects of CFA injection in the PEAP in SD and WKY rats (Day 11 post-injection). The ipsilateral hind paw was stimulated when the animal was in the dark side and the contralateral hind paw was stimulated when it was in the light side of the arena. (**A**) CFA-injected SD and WKY rats showed higher percentage positive response when stimulated with 60 g von Frey filament on the ipsilateral hind paw, compared to control counterparts. (**B and C**) Temporal profile of time spent and number of entries into the light side in SD and WKY rats during the 30-min trial. Neither SD-CFA nor WKY-CFA rats displayed increased (**B**) time spent or (**C**) number of entries into the light side over the course of the trial, compared to respective control counterparts. Data are presented in 5 min bins and expressed as mean ± SEM, *n* = 9–10/group. *p < 0.05 (SD-CFA vs. SD-control), ^+^*p *< 0.05 (WKY-CFA vs. WKY-control), ^#^*p *< 0.05 (WKY-control vs. SD-control). CFA, complete Freund's adjuvant; PEAP, place escape/avoidance paradigm; SD, Sprague–Dawley; WKY, Wistar–Kyoto.

We also measured the activity of the animals during the test. In the PEAP, an increase in activity (time spent or number of entries) in the light side reflects the degree of aversion to the noxious mechanical stimulation. Repeated measures ANOVA revealed a significant effect of time (F_3.823,133.806 _= 7.170, *p *< 0.001) and an overall significant effect of strain (F_1,35 _= 30.310, *p *< 0.001) on the time spent in the light side (Figure 4B). Further *post hoc* tests showed that the WKY-control rats spent less time in the light side of the arena, compared to SD-control rats, throughout the trial [T_0–30_ (in 5 min bins): WKY-control vs. SD-control, *p *< 0.05]. However, neither SD-CFA nor WKY-CFA rats spent more time in the light side, compared to respective control counterparts, at any of the time points during the trial.

In addition, all experimental groups exhibited a progressive decrease in the number of entries made into the light side of the arena (Figure 4C). Repeated measures ANOVA revealed a significant effect of time (F_3.379,118.263 _= 55.012, *p *< 0.001) and time x strain interaction (F_3.379,118.263 _= 2.657, *p *< 0.05) in tests of within-subjects effects on number of entries into the light side. Significant overall effects of strain (F_1,35 _= 40.573, *p *< 0.001), CFA (F_1,35 _= 13.277, *p *< 0.01), and strain x CFA interaction (F_1,35 _= 5.232, *p *< 0.05) were observed in tests of between-subjects effects on light side entries. *Post hoc* tests showed that the WKY-control rats made fewer entries into the light side, compared to SD counterparts [T_0–30_ (in 5 min bins): WKY-control vs. SD-control, *p *< 0.05] throughout the trial. Also, the SD-CFA rats entered less into the light side, compared to SD-control rats [T_0–30_ (in 5 min bins): SD-CFA vs. SD-control, *p *< 0.05]; however, no such effect of CFA injection was observed in the WKY rats.

### 3.4. CFA did not induce anxiety-related behavior in SD and WKY rats

**Figure 5:**
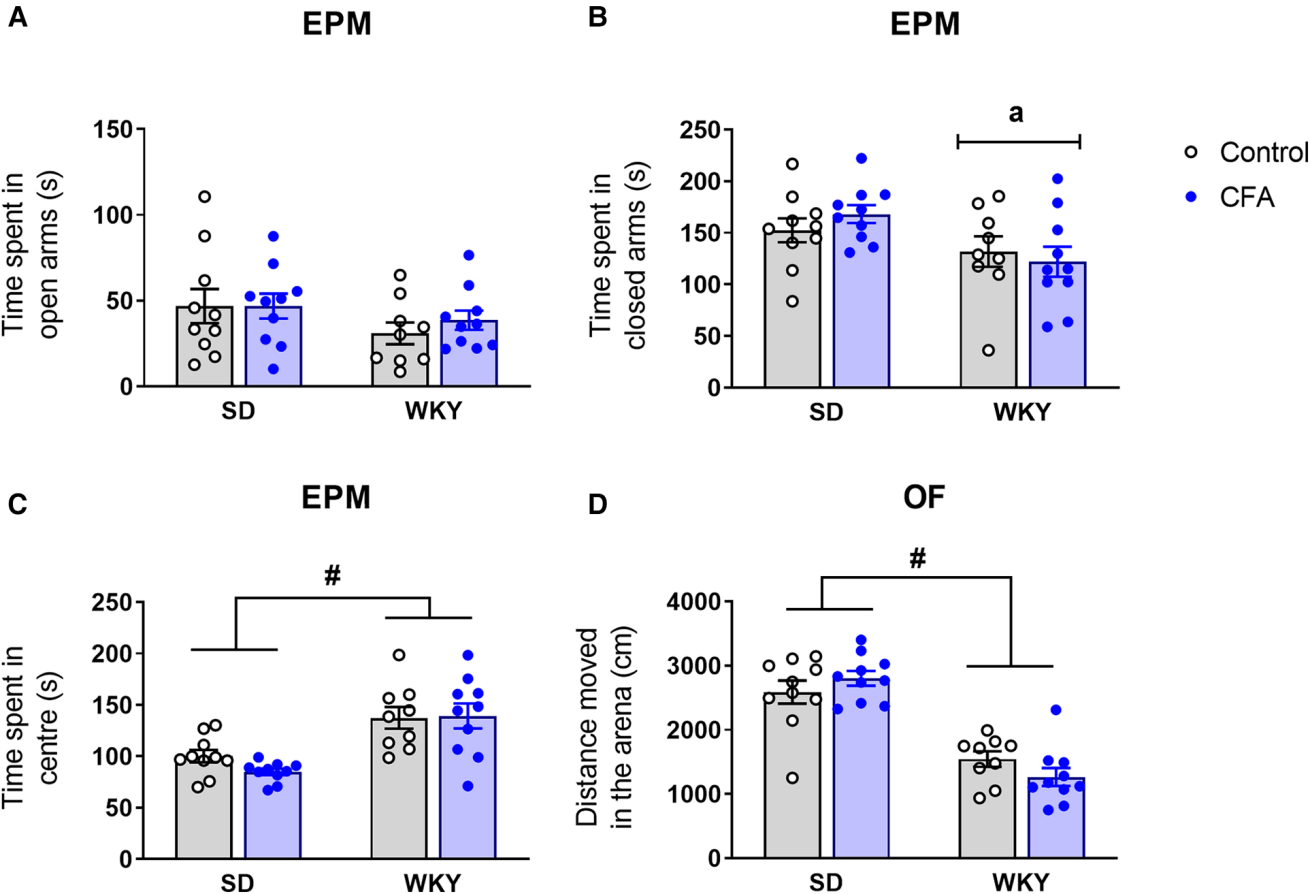
Effects of CFA injection on (**A–C**) anxiety-related behavior in the EPM and (**D**) general locomotor activity in the OF test in SD and WKY rats (Day 13 post-injection). (**A**) There was no significant strain difference in time spent in open arms. CFA injection did not affect time spent in open arms in either strain. Overall, WKY rats spent less time in the closed arms (**B**) but more time in the center (**C**) of the maze, compared to SD counterparts. (**D**) WKY rats displayed reduced distance moved in the OF, compared to SD rats. CFA injection did not affect distance moved in either strain. Data are expressed as mean ± SEM, *n* = 9–10/group. a = main effect of strain, *p *< 0.05; ^#^*p *< 0.05 (WKY vs. SD counterpart). CFA, complete Freund's adjuvant; EPM, elevated plus maze; OF, open field; SD, Sprague–Dawley; WKY, Wistar–Kyoto.

On Day 13 post-injection, anxiety-related behavior was assessed in SD and WKY rats using two behavioral tests – EPM and OF. In the EPM test, two-way ANOVA revealed that there were no significant effects of strain, CFA, and strain x CFA interaction on time spent in the open arms (Figure 5A). A significant main effect of strain (F_1,38 _= 7.072, *p *< 0.05) was observed on time spent in the closed arms but further *post hoc* analysis revealed that there were no differences between relevant experimental groups (Figure 5B). Activity in the center zone that connects the open and closed arms of the maze was also analyzed. Two-way ANOVA showed a significant main effect of strain (F_1,38 _= 29.270, *p *< 0.001) on time spent in the center zone. *Post hoc* analysis revealed that overall the WKY rats spent more time in the center of the maze compared to SD rats (WKY-control vs. SD-control, *p *< 0.05; WKY-CFA vs. SD-CFA, *p *< 0.05; Figure 5C).

Following EPM test, the animals were exposed to the OF test. Two-way ANOVA showed that there were no significant differences between the groups for time spent in the center of the arena [strain: F_1,38 _= 1.513, *p *= 0.227, CFA: F_1,38 _= 0.001, *p *= 0.994, strain x CFA: F_1,38 _= 0.890, *p *= 0.352; data not shown).

### 3.5. CFA did not alter locomotor activity in SD and WKY rats

General locomotor activity of the animals was also assessed in the OF arena. Two-way ANOVA revealed a significant main effect of strain (F_1,38 _= 71.284, *p *< 0.001) on distance moved in the OF arena (Figure 5D). *Post hoc* analysis showed that the WKY rats exhibited reduced distance moved in the OF compared to SD rats, indicating their characteristic hypolocomotor trait in a novel environment. In addition, CFA injection did not affect distance moved in the arena in either strain.

### 3.6. WKY rats displayed social avoidance behavior compared to SD rats

**Figure 6:**
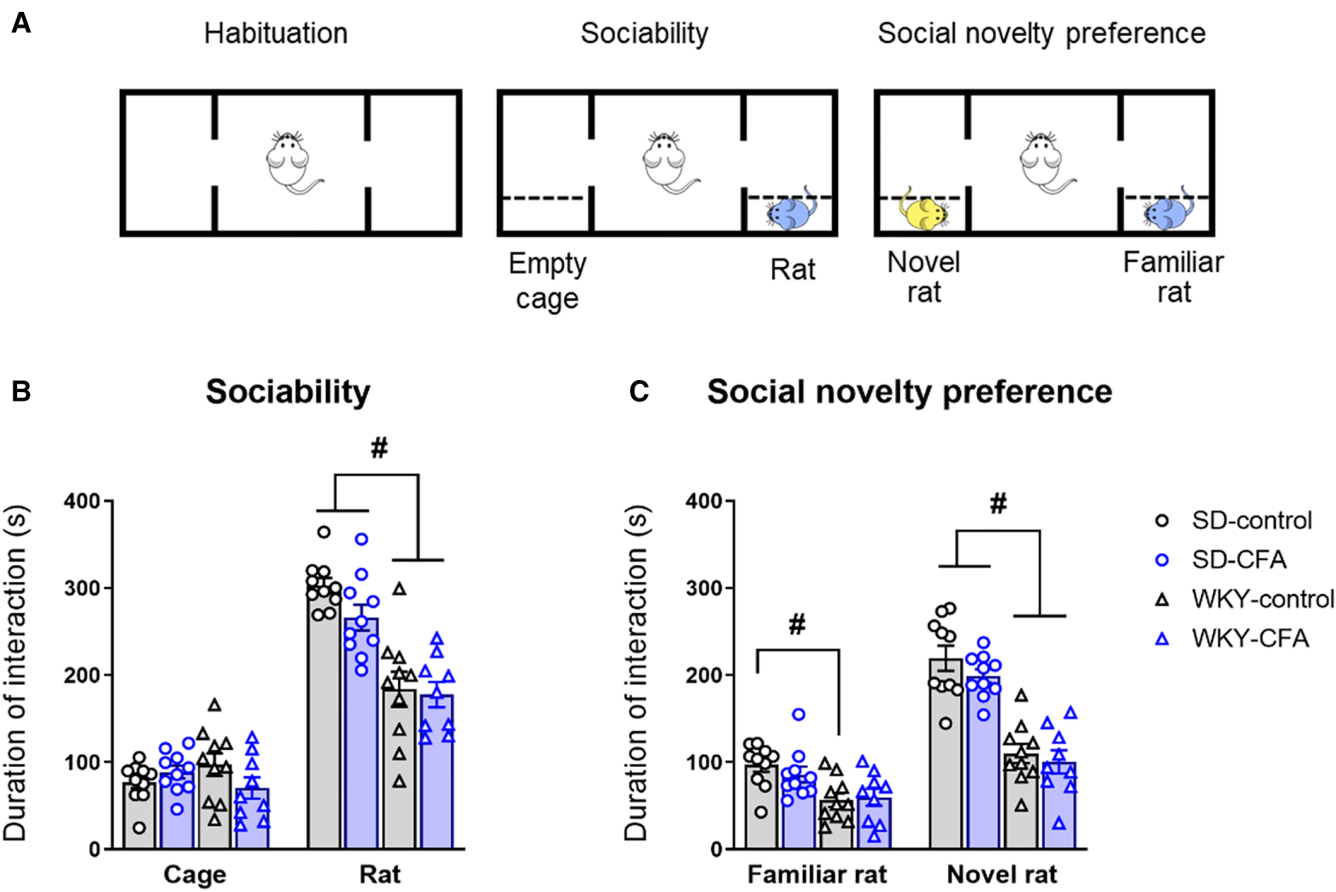
Effects of CFA injection on social behavior and social memory in SD and WKY rats in the 3-CST (Day 15 post-injection). (**A**) Schematic diagram of the time course of 3-CST. The test consists of three consecutive 10-min sessions where the test animal can freely explore the empty arena (habituation), an empty cage or a novel rat (sociability), and a second novel rat and the now familiar rat (social novelty preference). (**B**) In the sociability phase, WKY rats overall spent less time interacting with the conspecific rat (social stimulus), compared to SD rats. (**C**) In the social novelty preference phase, WKY rats overall spent less time interacting with the novel conspecific rat, compared to SD rats. CFA injection did not affect sociability or social novelty preference in either strain. Data are expressed as mean ± SEM, *n* = 9–10/group. ^#^*p *< 0.05 (WKY vs. SD counterpart). CFA, complete Freund's adjuvant; 3-CST, three chamber sociability test; SD, Sprague–Dawley; WKY, Wistar–Kyoto.

The 3-CST was used to assess social motivation (sociability) and social memory (novelty preference) of the test animal on Day 15 post-injection (Figure 6A). In the sociability phase, the test rat has a choice to interact with the empty cage (object, non-social stimulus) or the novel rat (social stimulus). As shown in Figure 6B, both SD and WKY rats displayed a preference for the novel rat over the empty cage. There were no significant effects of strain, CFA, and strain x CFA interaction on time spent exploring the empty cage. Two-way ANOVA did reveal a significant main effect of strain only (F_1,38 _= 47.328, *p *< 0.001) on time spent interacting with the rat in the arena. *Post hoc* analysis showed that the WKY rats, irrespective of CFA treatment, spent less time exploring the novel rat compared to SD counterparts (WKY-control vs. SD-control, *p *< 0.001; WKY-CFA vs. SD-CFA, *p *< 0.05; Figure 6B). This strain-related effect on social interaction remained significant after controlling for the covariate distance moved (ANCOVA: F_1,38 _= 24.131, *p *< 0.001).

Next, in the social novelty preference phase, the test rat is given a choice to interact with the then familiar rat or another novel rat introduced in the arena. Two-way ANOVA revealed a significant main effect of strain (F_1,38 _= 14.829, *p *< 0.001) on time spent exploring the familiar rat. *Post hoc* analysis showed that WKY-control rats spent less time exploring the familiar animal compared to SD counterparts (WKY-control vs. SD-control, *p *< 0.05; Figure 6C). Two-way ANOVA also revealed a significant main effect of strain (F_1,38 _= 77.556, *p *< 0.001) on time spent exploring the novel animal. Further *post hoc* analysis indicated that the WKY rats, regardless of CFA injection, spent less time exploring the novel animal in the arena, compared to SD counterparts (WKY-control vs. SD-control, *p *< 0.001; WKY-CFA vs. SD-CFA, *p *< 0.001; Figure 6C). This strain-related effect on social novelty preference was retained after adjusting for the covariate distance moved (ANCOVA: F_1,38 _= 58.660, *p *< 0.001).

### 3.7. WKY rats exhibited an overall reduced exploration of novel object compared to SD rats

**Figure 7:**
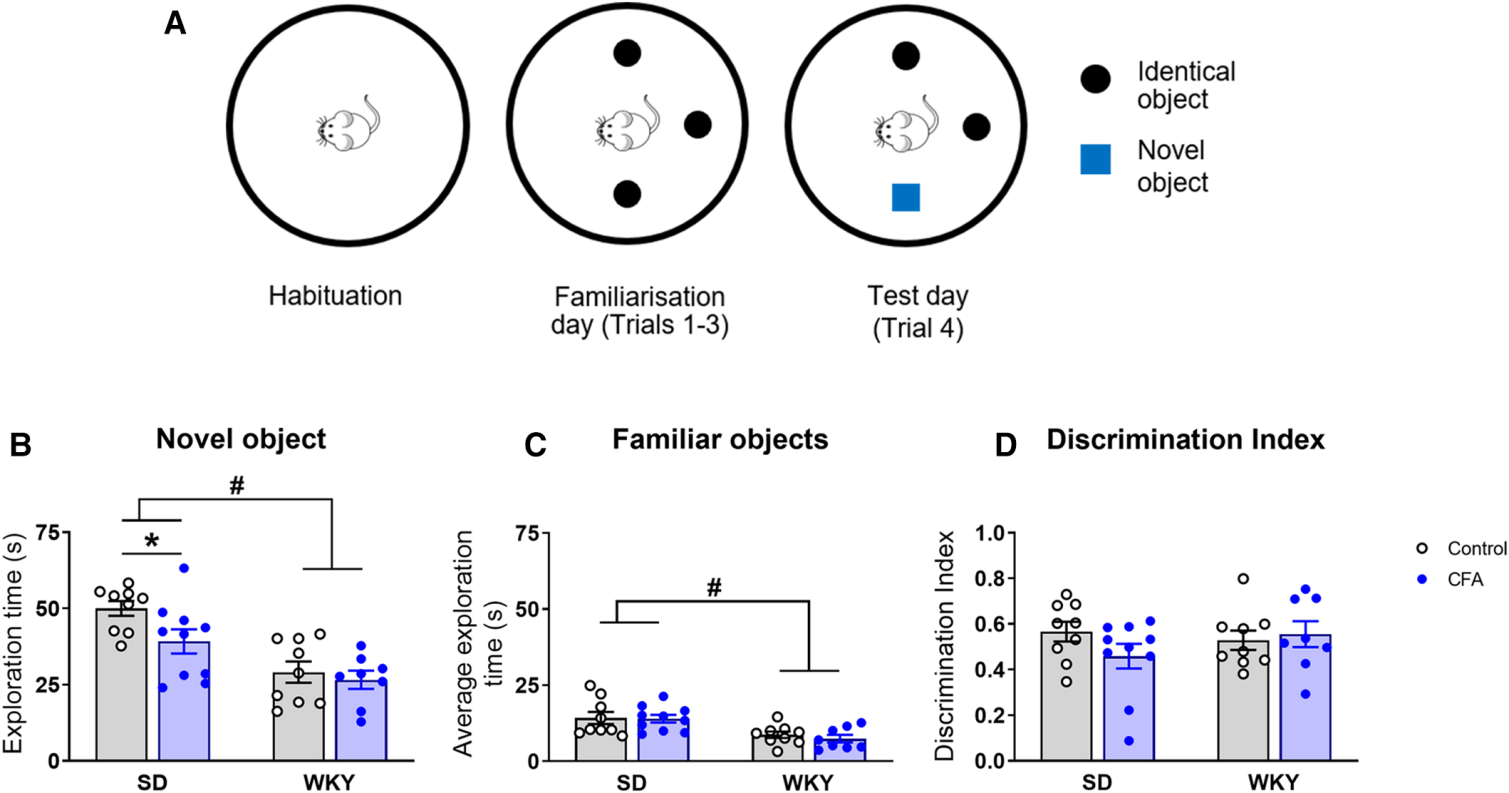
Effects of CFA injection on object recognition memory in SD and WKY rats in the NOR test (Day 18 post-injection). (**A**) Schematic diagram of the time course of NOR test. The test consists of 3 sessions on consecutive days: habituation (exploration of the empty arena), familiarisation (three trials, 5 min each, for exploration of three identical objects with 5 min inter-trial interval), and test (one trial of 5 min for exploration of familiar and novel objects). (**B**) Overall, WKY rats spent less time exploring the novel object in the arena, compared to SD rats. SD-CFA rats spent less time exploring the novel object than control counterpart. (**C**) WKY rats also spent less time exploring the familiar objects in the arena on the test day, compared to SD rats. (**D**) Discrimination index was similar between SD and WKY rats irrespective of CFA injection. Data are expressed as mean ± SEM, *n* = 8–10/group. *p < 0.05 (SD-CFA vs. SD-control), ^#^*p *< 0.05 (WKY vs. SD counterpart). CFA, complete Freund's adjuvant; NOR, novel object recognition; SD, Sprague–Dawley; WKY, Wistar–Kyoto.

The NOR test was employed to assess object recognition memory in SD and WKY rats on Day 18 post-injection (Figure 7A). Two-way ANOVA revealed significant main effects of strain (F_1,35 _= 26.001, *p *< 0.001) and CFA (F_1,35 _= 4.271, *p *< 0.05) on time spent exploring the novel object in the arena. Further *post hoc* test showed that the SD-CFA rats spent less time exploring the novel object, compared to control counterparts (SD-CFA vs. SD-control, *p *< 0.05; Figure 7B). The WKY rats, regardless of CFA treatment, spent less time exploring the novel object in comparison to SD rats (WKY-control vs. SD-control, *p *< 0.05; WKY-CFA vs. SD-CFA, *p *< 0.05; Figure 7B). This strain-related effect on novel object exploration remained significant after controlling for the covariate distance moved (ANCOVA: strain effect F_1,35 _= 5.851, *p *< 0.05).

In addition, two-way ANOVA revealed a significant main effect of strain only (F_1,35 _= 17.102, *p *< 0.001) on time spent exploring the familiar objects in the arena. *Post hoc* analysis indicated that there was no difference in the time spent exploring the familiar objects between control and CFA-injected SD rats (Figure 7C). The WKY rats, regardless of CFA treatment, spent less time interacting with the familiar objects, compared to SD counterparts (WKY-control vs. SD-control, *p *< 0.05; WKY-CFA vs. SD-CFA, *p *< 0.05; Figure 7C). This strain-associated effect on familiar object exploration was not evident after adjusting for the covariate distance moved.

Next, we evaluated the relative exploration of novel vs. familiar objects in the animals using discrimination index (Figure 7D). Two-way ANOVA failed to reveal any significant effect of strain, treatment, or their interaction on discrimination index.

### 3.8. Spatial memory impairment in the WKY rats compared to SD rats

**Figure 8:**
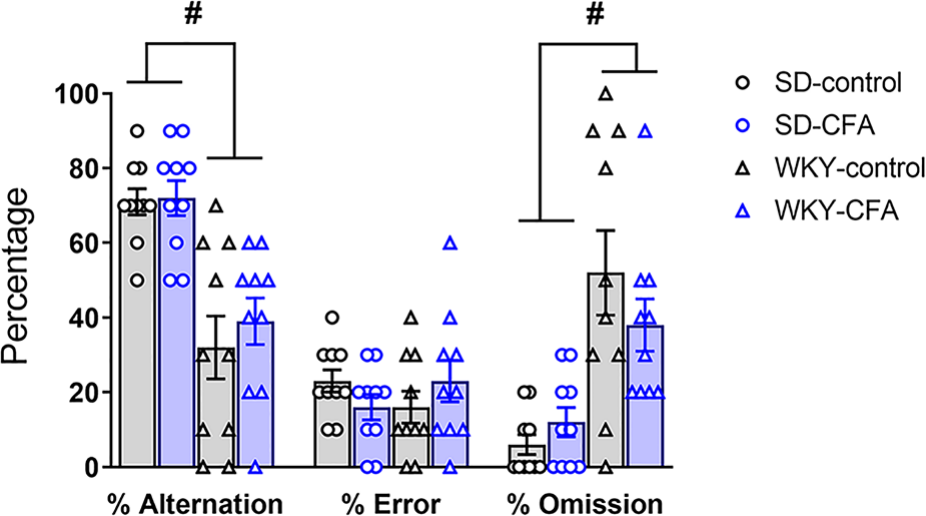
Effects of CFA injection in SD and WKY rats in the T-maze test of spatial memory (days 20 and 21 post-injection). Overall, WKY rats displayed lower percentage alternation of goal arms and higher percentage of trial omission, compared to SD counterparts. There was no difference in the percentage error in alternation of goal arms between SD and WKY rats. Also, CFA injection did not affect spatial memory in either strain in the T-maze test. Data are expressed as mean ± SEM, *n* = 10/group. ^#^*p *< 0.05 (WKY vs. SD counterpart). CFA, complete Freund's adjuvant; SD, Sprague–Dawley; WKY, Wistar–Kyoto.

Spatial memory was evaluated in SD and WKY rats using the T-maze task on Days 21 and 22 post-injection. Two-way ANOVA showed a significant main effect of strain (F_1,39 _= 36.167, *p *< 0.001) on percentage alternation (correct arm choice). *Post hoc* analysis showed that the WKY rats, irrespective of CFA injection, exhibited lower alternation of arms in the choice phase, compared to SD counterparts (WKY-control vs. SD-control, *p *< 0.05; WKY-CFA vs. SD-CFA, *p *< 0.05; Figure 8). There were no significant effects of strain, CFA, and strain x CFA interaction on percentage error in alternation (incorrect arm choice) in the ANOVA (Figure 8). In addition, two-way ANOVA revealed a significant main effect of strain (F_1,39 _= 26.036, *p *< 0.001) on percentage omission of trials (failure to choose an arm). *Post hoc* analysis indicated that the WKY rats, regardless of CFA injection, failed higher number of times in choosing an arm, compared to SD rats (WKY-control vs. SD-control, *p *< 0.05; WKY-CFA vs. SD-CFA, *p *< 0.05; Figure 8). CFA injection did not affect percentage arm alternation or trial omission in both SD and WKY rats.

## 4. Discussion

**Figure 9:**
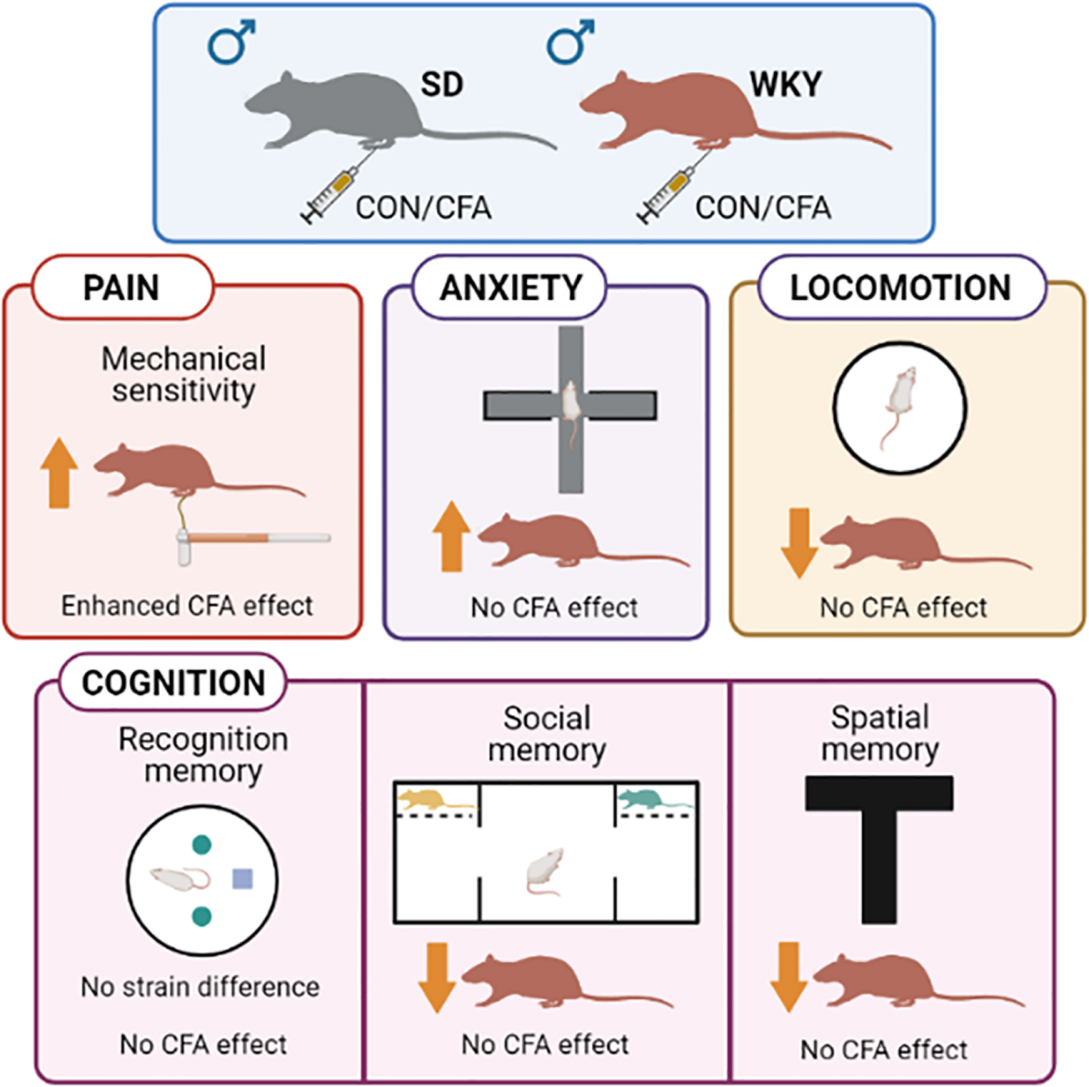
Summary of strain differences between WKY and SD rats in tests of pain, anxiety, and cognition-related behaviors. Schematic created with BioRender.com. CFA, complete Freund's adjuvant; CON, control, SD, Sprague–Dawley; WKY, Wistar–Kyoto.

To our knowledge, this is the first detailed and concurrent exploration of persistent inflammatory pain behavior (both sensory and affective aspects), anxiety-related behavior, and cognition (across multiple domains: social, object recognition, and spatial memory) in the WKY rat strain. Broadly, our results (Figure 9) indicate exacerbated CFA-induced alterations only in nociceptive behavior in the WKY rats but not in the other behavioral domains (negative affect, cognition) examined, contrasting somewhat with the initial hypothesis. We found that mechanical hypersensitivity was greater in the CFA-injected WKY rats than SD counterparts. In the PEAP, both CFA-injected WKY and SD rats did not exhibit pain avoidance behavior. However, the WKY rats spent less time in the light side of the arena than SD rats. Moreover, the CFA-injected SD rats, but not WKY counterparts, had fewer entries into the light side. CFA injection did not induce anxiety-related behavior or locomotor deficits in either strain at the time point tested. Impairments in social behavior and memory as well as spatial memory were apparent in control, but not in CFA-injected, WKY rats. Finally, the WKY rats displayed reduced novel object exploration, but not object recognition memory, compared to SD rats.

Our results at baseline suggest enhanced mechanical sensitivity in the naïve WKY rats, compared to SD rats, in agreement with previous reports in the literature (40, 41). This increased sensitivity to mechanical stimuli in the WKY rats was also evident on the contralateral side. Following CFA injection into the hind paw, rats from both strains developed mechanical and heat hypersensitivity as expected. However, CFA induced greater mechanical hypersensitivity in WKY rats than SD rats in the von Frey test. This finding contrasts somewhat with the study of Hestehave et al. who reported that the WKY rats develop a less robust mechanical hypersensitivity (measured using the Randall–Selitto test) after CFA injection (42). The discrepancies in results could relate to the differing methodologies used to assess mechanical sensitivity and at different thresholds (i.e., nociceptive response due to a normally non-noxious stimulus vs. a noxious stimulus). In the present study, both WKY and SD rats developed similar hypersensitivity to noxious heat following CFA injection. Hence, it appears reasonable to suggest that the WKY rats show modality-dependent exacerbated nociceptive responding in the CFA model, indicating an influence of genotype on the sensory component of pain. Of note, such augmented nociceptive behavior in the WKY rat strain has been reported using different models of chronic pain (27, 28, 43).

The experience of pain is multidimensional comprising of sensory, affective, and cognitive components. We used the PEAP to assess the affective aspect of pain. The PEAP is based on a conflict between the natural tendency of the rodents to avoid light and the avoidance of noxious stimuli in the dark side (34). Several studies have reported that CFA-injected rats spend more time in the light side compared to control counterparts over the course of the test (12, 44). These results indicate that the animals tend to avoid the increased aversiveness induced by stimulating the injured (i.e., CFA-injected) paw. In our study, the SD rats showed CFA-induced mechanical hypersensitivity when the ipsilateral paw was stimulated in the dark side. Despite this, we found that the SD-CFA rats preferred to remain in the dark side over the course of the trial, in contrast to these published studies. An important difference between the previous reports and our experiment is the time point of conducting PEAP (24–48 h vs. Day 11 post-CFA), which may partly account for these discrepant findings. It appears that although CFA induces enhanced sensory response to noxious mechanical stimuli, the aversiveness of CFA-induced pain may differ over time (early vs. later), possibly due to compensatory or adaptive changes in an ongoing pain state. For instance, Wu and colleagues have examined pain aversion (place conditioning paradigm) and anxiety-related behavior (elevated zero maze test) in the CFA model (45). The authors have shown that CFA-induced aversion is highest in week 1 post-CFA and gradually attenuates over 4 weeks in SD rats, while anxiety-related behavior is only apparent at week 4 post-inflammation but not at earlier time points (45). These findings may partly explain our results and further support the contention that the manifestation of negative affect (as pain aversion or pain-associated anxiety-related behavior) in a persistent pain model may follow different time courses. Furthermore, we found that in the PEAP the WKY rats displayed an overall reduced activity (both duration and entries) in the light side, compared to SD rats. The WKY rats show reduced locomotor activity in the OF (also seen in the current study), compared to other rat strains including SD rats (17, 18, 21, 46, 47). Additionally, the WKY rats has been reported to exhibit a behaviorally inhibited phenotype in response to novel social and nonsocial challenges (19, 48, 49). Thus, the elevated anxiety level along with diminished locomotor activity of the WKY rats in a novel environment may, to some extent, account for their behavior observed in the PEAP in the present study.

In the tests to measure anxiety-related behavior, the WKY rats displayed an overall propensity to escape/avoidant behavior, compared to SD rats. We note that unlike previous reports the WKY rats in our study did not display classic anxiety-like behavior in the EPM or OF (e.g., reduced time spent in the open arms or center zone, respectively) (17, 18, 21, 50). Rather, we observed that the WKY rats spent considerable time in the center square of the EPM. At least two other groups have reported similar findings in the WKY rat strain (51, 52). Moreover, the WKY rats exhibited decreased activity in the novel aversive environment of the OF in line with previous reports (17, 18, 21, 53). Together, these findings indicate a certain degree of behavioral inhibition that may be related to elevated fear/anxiety level in the WKY rats. Additionally, we did not observe any CFA-induced anxiety-like behaviors in either strain unlike previous studies (45, 54, 55). Methodological difference in testing time points between our experiment and other reports (Day 13 vs. 3–4 weeks post-inflammation) may account for this discrepancy. Future studies could examine whether the WKY rats show exaggerated anxiety-like behaviors in the CFA model at later time points.

The present study also examined strain differences in multiple cognitive domains in the CFA model. In the 3-CST, the WKY rats, compared to SD counterparts, displayed reduced interaction with the novel rat relative to the cage, suggesting a propensity to inhibition to social cues as reported previously (52, 56). However, no strain difference was observed in the relative exploration of novel vs. familiar rat. Thus, the reduced social interaction displayed by the WKY rats is likely related to their social avoidance behavior, rather than a deficit in social recognition memory (57). In the NOR test, although the discrimination index was similar between the two strains, the WKY rats clearly exhibited reduced exploration of the novel object compared to SD rats. Others have also reported similar results with the WKY rats in the NOR paradigm (58–60). This observation suggests that the WKY rats may not have an impairment in object recognition memory *per se* but display an overall avoidance behavior to novelty (61) that is reflected in reduced object exploration times. In the T-maze test, the WKY rats exhibited reduced arm alternation compared to SD rats, suggesting a possible impairment in spatial memory in this rat strain (62). This reduced alternation displayed by the WKY rats could also be due to their increased failure in arm choice which further reflects their indecisive phenotype (52). It should be noted that all these cognitive tasks rely on locomotor behavior. Thus, alternatively it is possible that the hypolocomotor trait of the WKY rats influences these results. However, ANCOVA to adjust the locomotor effect indicates this to be unlikely. In this regard, some studies have reported that the WKY rats are not hypolocomotive in the rotarod test (63) or during home cage activity (18). These results suggest that the WKY rats may not have an overt deficit in motor activity, but that their deficiencies are unmasked in a novel (aversive) environment. It would be interesting to assess cognitive parameters in the WKY rats in a familiar environment [e.g., NOR task in the home cage (64)] to see if the deficits persist. Given that the WKY rats display behavioral inhibition and reduced exploratory behaviour in a novel environment, a familiar context could help to differentiate these traits and allow evaluation of more nuanced naturalistic behaviors. Furthermore, following CFA injection neither SD nor WKY rats exhibited impairment in the cognitive domains (object recognition, social, and spatial memory) examined in the current study. Reduced direct social interaction has been observed in SD rats on Day 28 after CFA injection (55). In addition, CFA-induced impairment in learning and memory has been reported in rodents in various paradigms like the Morris water maze, NOR, and Y-maze (31, 65, 66). However, methodological differences such as behavioral tasks used and test time points in these reports may account for the differences in results observed in our study. Hence, further studies are warranted to understand the impairments in specific cognitive domains associated with chronic inflammatory pain in the CFA model.

## 5. Conclusions

In summary, the results of this study indicate exacerbated mechanical but not heat hypersensitivity in the WKY rats vs. SD rats in the CFA model. Moreover, the manifestation of the affective component of CFA-induced pain may follow a different time course than the sensory component. Differences exist in the domains of social behavior and social and spatial memory between WKY and SD rats. The behaviorally inhibited trait of the WKY rats may affect performance in some of these cognitive parameters. The results do not provide evidence for CFA-induced anxiety-related behavior or impairments in object recognition, social, or spatial memory in either strain at the time points tested.

Taken together, the present study extends our understanding of the unique behavioral characteristics of the WKY rat strain as well as CFA-associated effects on different behavioral domains (pain, affect, and cognition) in both SD and WKY rats. Our findings emphasize that the WKY rat is particularly relevant for studying specific functional domains such as anxiety vulnerability, social withdrawal, learning deficit, and aberrant pain responding that contribute to comorbid chronic pain and affective disorders/cognitive dysfunction. Thus, the WKY rats will be a useful preclinical model to elucidate the mechanisms underlying the pathophysiology of these comorbid conditions and ultimately novel therapeutic strategies. Regarding the CFA model, it is reliable in studying genotype-dependent effects on nociceptive behavior. However, questions remain regarding the use of the CFA model to assess the effects of persistent inflammatory pain on negative affect and cognition. Though many studies have shown positive association in these measures (13, 45, 55, 67), the results are inconsistent (68–70) largely due to differences in methodology. Thus, further comprehensive investigation is necessary to understand the translational value of the CFA model in pain comorbidity.

## Data Availability

The raw data supporting the conclusions of this article will be made available by the authors, without undue reservation.
